# Studies on Corrosion Initiation in Reinforced Concrete Structures Using Ground-Penetrating Radar

**DOI:** 10.3390/ma18102308

**Published:** 2025-05-15

**Authors:** Wiktor Wciślik, Wioletta Raczkiewicz

**Affiliations:** Faculty of Civil Engineering and Architecture, Kielce University of Technology, Al. 1000-lecia PP 7, 25-314 Kielce, Poland; wiolar@tu.kielce.pl

**Keywords:** reinforced concrete, corrosion, non-destructive testing, laboratory test, ground-penetrating radar (GPR), durability

## Abstract

The present article describes an example of the use of ground-penetrating radar (GPR) to detect early stages of reinforcement corrosion. Two series of concrete samples with reinforcing bars were tested. The first series was reference samples (without corrosion). Samples of the second series were subjected to accelerated corrosion by immersing them in NaCl solution, while undergoing 120 freeze–thaw cycles. Unlike the commonly used electrochemical method of corrosion acceleration, in the studies discussed here, the corrosion processes were more similar to natural ones, taking into account the influence of changes in the structure of the cover under the influence of frost. GPR scanning of samples of both series indicated that all physical and chemical processes accompanying corrosion together caused a decrease in the amplitude of the reflected wave and an increase in its propagation time. The wave amplitude, due to the significant dispersion of results, was, however, a rather unreliable parameter. The wave propagation time was characterized by significantly better repeatability, which makes it a better measure of the progress of corrosion. In general, the GPR with a 2 GHz antenna proved to be an effective tool for diagnosing early stages of corrosion in reinforced concrete.

## 1. Introduction

### 1.1. Reinforcement Corrosion and Its Consequences

Reinforcement corrosion in concrete is a direct cause of reduced durability in reinforced concrete structures, leading to a loss of load-bearing capacity, stability, and serviceability conditions [1,2]. The initiation and development of corrosion in steel reinforcement bars within reinforced concrete elements are primarily influenced by environmental factors [3,4], including the effects of carbon dioxide [5,6] or chloride penetration into the concrete structure [7,8,9]. Additionally, this phenomenon can be intensified by frost [10]. These factors cause damage to the concrete cover—the layer of concrete that is intended to provide protective functions for the steel.

Its protective role arises from the fact that concrete has a highly alkaline pH (pH = 11.5–13.5), which leads to the formation of a passive layer (1–100 nm thick) on the surface of the reinforcement bars. This layer consists of iron oxides (Fe_2_O_3_ and Fe_3_O_4_) with very low ionic conductivity, protecting the reinforcement against corrosion [2]. However, if the pH of the concrete drops below 11.8 due to the presence of carbon dioxide in the air, the passive layer on the reinforcement is damaged, and the corrosion process may begin [11].

Another cause of corrosion is the action of chlorides, which, in the form of chloride ions (Cl^−^) dissolved in water, penetrate the pores of the concrete structure and can lead to pitting corrosion [12,13,14]. This is often associated with the use of deicing agents containing NaCl in winter and the cyclic freezing and thawing of moisture within concrete pores [10,15]. Notably, pitting corrosion can develop even at seemingly safe pH levels (pH > 11.8).

An indirect cause of potential future corrosion processes in reinforcement is the presence of other chemical factors, which, although not as common as those mentioned above, contribute to the deterioration of the concrete cover. These include the effects of sulfur compounds, inorganic and organic acids, and magnesium salts, which react with the components of hardened concrete, increasing its porosity and indirectly accelerating steel corrosion [16]. Another contributing factor can be mechanical damage, which leads to cracks and fractures in the concrete, creating pathways for environmental influences [17,18].

Although the causes of corrosion may vary, the process itself usually follows a similar pattern. It is an electrochemical process resulting from the physicochemical properties of both concrete and steel. A steel reinforcement bar acts as a first-type conductor, meaning it serves as an electron carrier. Concrete, due to its structure—comprising interconnected pores filled with liquid—allows ion transport via diffusion [2]. This creates a type of electrochemical cell, where the liquid filling the concrete pores acts as an electrolyte, and the steel reinforcement bar serves as an electrode immersed in the electrolyte.

When the passive layer protecting the reinforcement surface is damaged (typically due to concrete carbonation or chloride action), and the concrete pores contain liquid while oxygen is present, the corrosion process begins. At the anode, iron undergoes oxidation:

2Fe → 2Fe^2+^ + 4e^−^ (1)

and at the cathode, the reduction process occurs:

2H_2_O + O_2_ + 4e^−^ → 4OH^−^ (2)

In the next stage of the corrosion process, iron ions react with OH^−^ ions, forming soluble iron hydroxide:

Fe^2+^ + 2OH^−^ → Fe(OH)_2_ (3)

and then, in the presence of oxygen:

2Fe(OH)_2_ + 1/2O_2_ → 2FeOOH + H_2_O (4)

**Figure 1:**
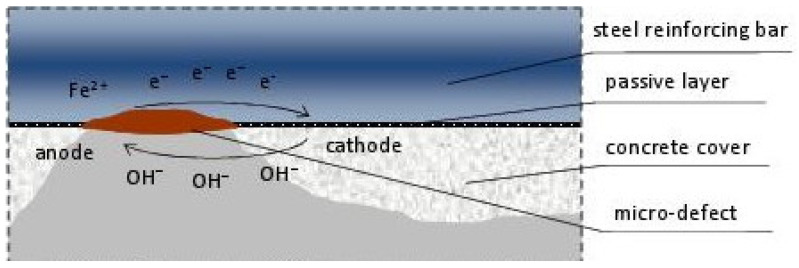
Schematic diagram of the electrochemical corrosion process of reinforcement in concrete.

This is schematically shown in Figure 1.

As a result of the ongoing processes, corrosion products, commonly known as rust, are formed, which is a mixture of phases: Fe(OH)_3_, Fe_2_O_3_, Fe_2_O_3_·H_2_O, and magnetite. These products accumulate on the surface of the reinforcement, causing internal stresses in the concrete, delamination from the reinforcement, and finally, detachment.

### 1.2. Methods of Corrosion Diagnosis

Since in reinforced concrete structures, the concrete cover protecting the reinforcement can mask the development of corrosion, assessing its advancement is often significantly difficult. However, early detection of this process allows for effective inhibition or at least the slowing down of corrosion. Therefore, various research methods used to assess the condition of reinforced concrete elements in this regard are of great importance. These methods typically include tests on concrete to evaluate its pH (degree of carbonation) and chloride content, as well as assessments of the degree of corrosion of the reinforcement [19,20].

Concrete testing can be performed directly on the structure using simple colorimetric tests or through laboratory evaluation of material samples taken from the structure, based on chemical, thermal, X-ray structural analysis, or scanning electron microscopy [21,22,23]. These tests are generally qualitative and mainly serve to determine the causes of corrosion, but they do not provide much information about the degree of corrosion of the reinforcement across the entire element or forecast its rate.

More valuable for assessing the degree of corrosion of reinforcement are so-called electrochemical research methods. These involve the analysis of measurements of certain electrical quantities, which, when compared to critical values, allow for determining the probability of corrosion in the studied element, assessing its advancement, and estimating its rate [15,24]. Such tests can be performed in laboratories, which involves taking samples and thus damaging the structural elements. However, they can also be carried out directly on the structure by applying so-called semi-destructive methods. Naturally, these methods are preferred over laboratory methods because they can be applied directly to the structure, quickly, and without the need to shut down its operation. Semi-destructive electrochemical methods only require a small section of concrete to be removed from the element to connect the measuring equipment to the reinforcement bars being tested. These methods are divided into simple and advanced types. Simpler methods include measuring the stationary potential of reinforcement [25] or measuring the resistivity of the concrete cover [26], performed on the concrete surface. These are considered qualitative measurements that effectively determine the level of the concrete cover’s tightness and its ability to conduct ions via diffusion. More advanced methods include measurements of the corrosion current density, which make it possible to determine the degree of surface loss of the bar due to corrosion products and, based on this, estimate the so-called corrosion activity of the reinforcement and forecast its development rate over time [24]. Since the methods described require polarization of the reinforcement bar being assessed (i.e., stimulating current flow), they are known as polarization methods. Depending on how the disturbance is initiated, they are divided into electrochemical impedance spectroscopy (where the disturbance is caused by alternating current over a wide frequency range) [27], linear polarization resistance (where the disturbance is generated by applying a linearly varying potential) [28], and galvanostatic pulse method (where the disturbance is generated by a current of appropriate intensity) [29].

Late stages of corrosion development and the associated delamination of the concrete–reinforcement interface can be effectively diagnosed by means of the analysis of induced elastic waves [30,31,32,33,34,35].

Recently, the use of ground-penetrating radar (GPR) technology has become increasingly popular for corrosion assessment [36].

The present article investigates the possibility of detecting early stages of corrosion development in reinforced concrete structure elements using ground-penetrating radar.

## 2. Related Work

### 2.1. Principle of GPR Operation

The ground-penetrating radar (GPR) method belongs to the group of geophysical methods and is primarily used for detecting objects hidden within the tested medium.

**Figure 2:**
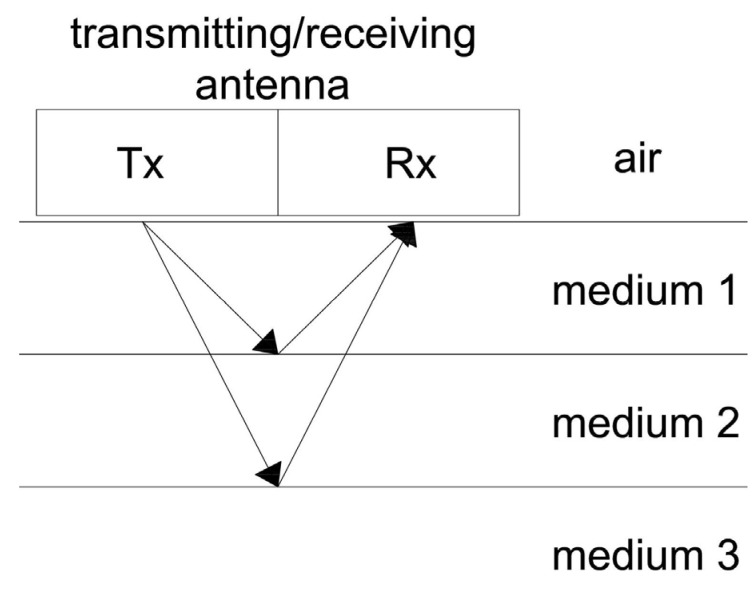
Ground-penetrating radar (GPR) operation principle.

There are different variants of how measurements are conducted, but in structural engineering investigations, reflective profiling is almost always used. A schematic diagram of this type of measurement is shown in Figure 2. The transmitting and receiving antennas are simultaneously moved across the surface of the studied material. The transmitting antenna sends an electromagnetic pulse into the medium. This signal, upon encountering an object/obstacle, is reflected and recorded by the receiving antenna. The wave reflected from the object is characterized by particularly high amplitude, and its analysis allows for assessment of the internal structure of the tested medium.

A condition that must be met for the detection of an object is the presence of a contrast in the dielectric constant (ε_r_) between the medium and the hidden object. In relation to concrete (ε_r_ in the range of 9–12), significant contrasts are provided by air (ε_r_ = 1), water (ε_r_ = 81), and metal (ε_r_ = ∞, perfect conductor). This means that GPR is a useful diagnostic tool for detecting objects in concrete, such as reinforcement bars, voids (filled with water or air), as well as cracks, delaminations, and material losses.

**Figure 3:**
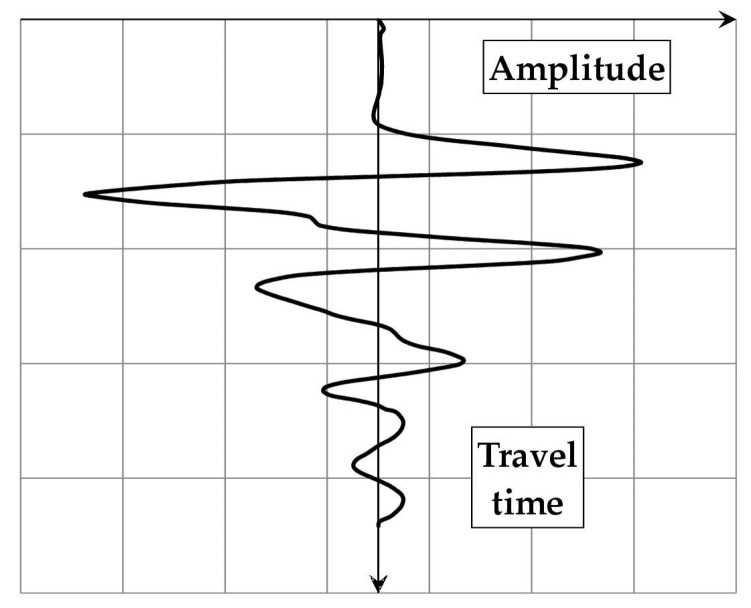
An exemplary GPR trace (A-scan).

The primary result of a GPR measurement is a single trace (A-scan, Figure 3), in which the amplitude of the reflected wave is plotted against the time of its propagation from the antenna to the medium and back. As mentioned before, a local increase in amplitude is generally associated with the reflection of the wave from an object.

The amplitude–time graph presented in Figure 3 is, however, of limited use when attempting to locate the detected object. Therefore, for practical purposes, the time scale is transformed into a depth scale, according to the following formula:

(5) $$s=\frac{vt}{2}$$

where s—target depth, t—wave two-way travel time, and v—wave velocity.

As indicated by the formula above, obtaining the depth scale requires knowledge of the wave propagation speed in the tested medium. This speed can be determined in various ways, with the most reliable being the measurement of the travel time of the wave between the antenna and an object located at a known depth (e.g., exposed reinforcement in a concrete structure). In the case of concrete, the wave speed typically oscillates around 10 cm/ns.

**Figure 4:**
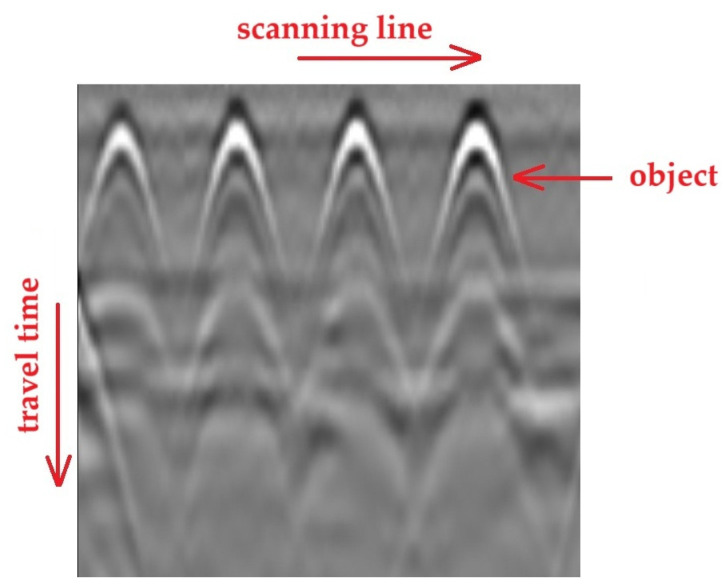
GPR record in the form of a B-scan.

A combination of adjacent traces (A-scans) allows for the creation of a B-scan (Figure 4), where the vertical axis, as previously, represents the time/depth scale, the horizontal axis corresponds to the antenna’s travel path, and the amplitude values are represented using the adopted color scale.

### 2.2. Areas of GPR Application in Construction

Although the initial applications of GPR were in geology, mining, and mineral exploration, the versatility of the method has allowed its use in other areas over the years. With the advent of high-frequency antennas, GPR has been implemented in various branches of construction, primarily in road construction [37,38], railway [39,40], and bridge construction [41,42], as well as in industrial and residential buildings.

As seen above, GPR is primarily used for non-destructive inventorying of the internal structure of members. However, the sensitivity of the electromagnetic wave to the physical properties of the tested medium has led to increased research on evaluating certain physical parameters of concrete based on the analysis of GPR wave parameters. This is a topic of significant practical importance, as material property assessments can be performed simultaneously with GPR scanning to assess the internal structure of construction elements.

In terms of evaluating the properties of concrete, the most common parameters studied are water content [43,44,45,46,47], chloride concentration, and both parameters simultaneously [48,49,50,51]. Authors agree that as the water and chloride content in concrete increases, the amplitude of the reflected wave decreases, as does its propagation speed.

### 2.3. Analysis of Corrosion Using GPR

In recent years, many studies have focused on the issue of detecting reinforcement corrosion using GPR [36,52,53]. However, the published results vary regarding the influence of corrosion products on the parameters of the reflected radar wave. It is assumed that this may be due to different phenomena occurring at various stages of the corrosion process, such as initiation (the concentration of chloride ions around the rebar, leading to a decrease in amplitude), the formation of microcracks, and the migration of corrosion products (increasing amplitude as a result of increased reflection surface), as well as delamination of the concrete cover (subsequent decrease in amplitude due to signal scattering around the crack area).

In most published studies, accelerated corrosion is typically induced by the application of current. Tesic et al. [54] investigated the effect of corrosion on the amplitude of the reflected wave. The samples were subjected to accelerated corrosion by immersing them in a sodium chloride solution and applying an impressed current technique (IC). For samples fully immersed in the NaCl solution, the corrosion products were uniformly distributed around the rebar, resulting in a reduction in the reflected GPR wave amplitude. In contrast, samples submerged in a solution below the rebar showed no corrosion products near the concrete cover, so the reflected wave amplitude did not change due to corrosion.

A decrease in wave amplitude and propagation speed was also observed by other authors [55,56], who suggested that this could be due to the presence of moisture in the tested samples. Regardless of the moisture content, it is generally believed that the decrease in amplitude is caused by signal scattering, which occurs due to the formation of microcracks in the concrete, corrosion products, and the roughening of the rebar surface [36].

Contrary to these results, Raju et al. [57] observed an increase in the amplitude of the reflected wave as corrosion progressed. Furthermore, as described by Zaki et al. [58], during GPR testing at various stages of corrosion development, the reflected wave amplitude did not show a clear trend, either increasing or decreasing as the corrosion process advanced. Liu et al. [59] emphasized the influence of the wave polarization direction on measurement accuracy.

The complexity of the corrosion process and its associated ambiguous effect on the reflected wave parameters was also noted by Hong et al. [60], who used numerical analysis to investigate the issue. They found that both the corrosion-induced reduction in rebar diameter and the formation of cracks in the concrete surrounding the rebar led to a decrease in the reflected wave amplitude. On the other hand, when cracks were filled with corrosion products, the amplitude increased. A decrease in the reflected wave amplitude due to corrosion-induced thinning of the rebar was also experimentally confirmed in a study by Eisenmann et al. [61].

The identification of the corrosion progress level using GPR data and machine learning algorithms was discussed by Wong et al. [62] and Faris et al. [63].

As seen from the above, despite numerous studies, a clear and unified understanding of how corrosion changes in rebar affect the reflected GPR wave properties has not yet been achieved. This seems to be due to the complexity of the phenomena occurring during corrosion. The oxidation of reinforcement steel is accompanied by factors such as the presence of chlorides and water in the concrete pores, as well as the formation of small cracks. Many of the studies published to date do not take into account the effects of cyclic freezing and thawing, which undoubtedly accompany the development of corrosion in concrete, such as in bridges.

In this article, we attempt to characterize the impact of corrosion phenomena on the GPR wave parameters, considering the changes in the concrete structure due to cyclic freezing and thawing.

## 3. Materials and Methods

**Figure 5:**
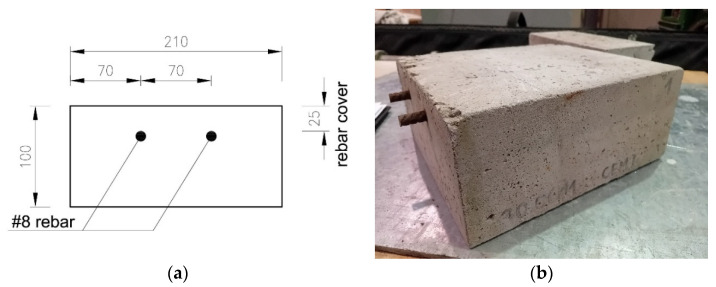
Samples used for the tests: (**a**) sketch (dimensions in [mm]), (**b**) sample view.

In order to assess the possibility of detecting corrosion initiation using GPR, two series of six concrete samples were prepared, as shown in Figure 5.

The samples were in the form of cubes with dimensions 228 × 210 × 100 mm^3^. Inside each cube, two rebars with a diameter of 8 mm were embedded, maintaining a constant cover thickness of 25 mm.

Concrete was prepared according to the recipe described in Table 1:

All the samples from both series were prepared in the same way and under identical laboratory conditions. The samples were demolded one day after casting and then cured for the next 14 days. In the next stage, the samples were stored in a laboratory hall at a temperature of approximately 20 °C until they were completely dry. The samples from the first series were kept in conditions that excluded degradation. The samples from the second series were subjected to accelerated corrosion by chloride action and freezing cycles. For this purpose, the samples were immersed in a 3% NaCl solution and placed in a frost resistance test chamber with an automatically controlled test program. The samples were subjected to 120 freezing and thawing cycles in the temperature range from +18 °C to −18 °C. One cycle lasted about 8 h, so usually 3 cycles were performed per day. During the freezing cycles, the samples were completely immersed in the solution. During the freeze–thaw cycles, the ends of the reinforcement bars protruding from the samples were isolated and did not come into contact with the solution. The protruding ends of the bars were tightly wrapped with several layers of insulating tape (with parameters that ensure its effectiveness at temperatures from −40 °C to +105 °C and in an aggressive environment) and sealed with grease. The method of initiating corrosion was therefore very similar to the conditions experienced by real structural elements, such as those in bridges. After completing the cycle, the samples were dried until their weight stabilized.

**Figure 6:**
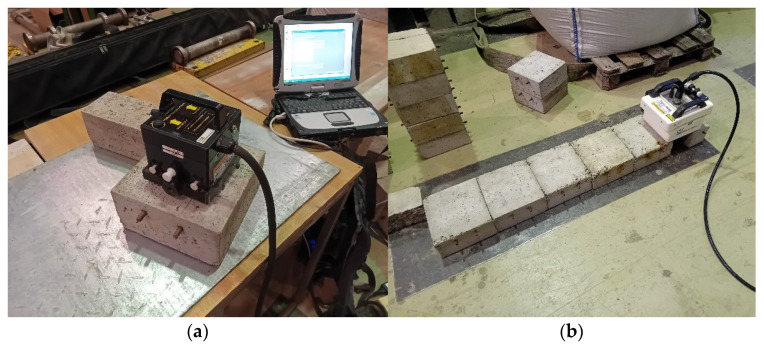
Scanning of concrete samples using GPR: (**a**) 2 GHz antenna, (**b**) 900 MHz antenna.

Subsequently, both series of samples (Series 1—samples with no signs of degradation, and Series 2— subjected to accelerated corrosion) were scanned using ground-penetrating radar (GPR, Figure 6).

An IDS Aladdin GPR system with ground-coupled antennas of 2 GHz and 900 MHz frequencies was used. The scanned samples were placed on a metal plate. The measurement involved passing the antenna through the center of the sample perpendicularly to the reinforcement bars. For each sample, thirty passes were made in two directions. Considering that 6 samples were prepared from each series (non-corroded and corroded samples), the measurements for each series included 180 results.

The assessment of the reinforcement corrosion progress in the tested samples was also analyzed using the electrochemical polarization galvanostatic pulse method (GPM) using the GP-5000 GalvaPulse^TM^ apparatus [29]. The obtained results, including the values of the stationary potential and the density of the corrosion current intensity of the tested bars, as well as the resistivity of the concrete cover, were analyzed in relation to certain limit values. Detailed information on this subject can be found in publications [6,10], which constitute a broader summary of the results of tests performed using the electrochemical method. The results obtained at that time indicated a significant increase in the corrosive activity of the reinforcement in samples exposed to 3% NaCl and freezing cycles in relation to the samples left in a non-aggressive environment. According to the markings used in the GPM method, the corrosive activity of the tested reinforcement increased from “unpredicted” to “moderate corrosive activity”.

## 4. Results and Discussion

**Figure 7:**
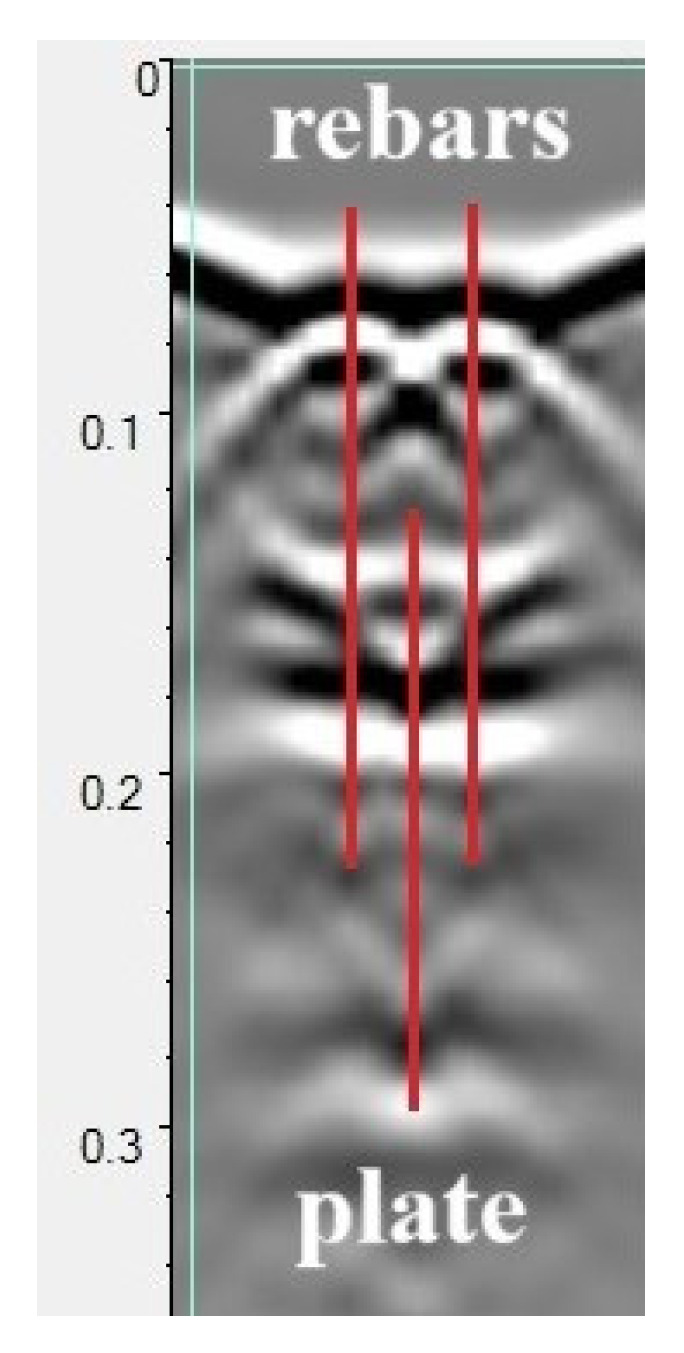
A sample radargram recorded during the scanning of the sample. The outer lines indicate the A-scans on which the wave reflection from the reinforcing bars was analyzed, the center line the A-scan on which the reflection from the sheet metal under the sample was determined.

The A-scans recorded directly above the reinforcement bars and in the middle of the distance between them were analyzed, as shown in Figure 7. The external vertical lines represent the paths (A-scans) used to analyze the parameters of the wave reflected from the reinforcement bars. The central line represents the path used to analyze the wave reflected from the plate beneath the sample. In turn, the parameters of the surface wave were read from all three A-scans marked below.

**Figure 8:**
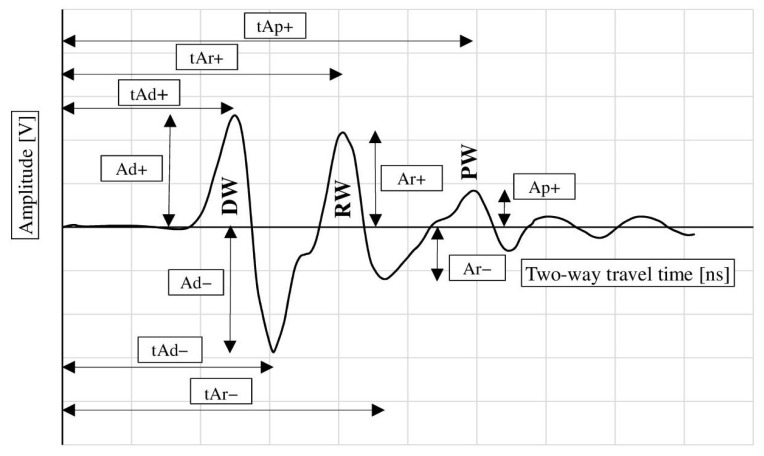
Measured GPR wave parameters: DW—surface wave, RW—wave reflected from the reinforcement, PW—wave reflected from the metal plate placed under the sample.

Figure 8 illustrates the wave parameters analyzed. These include the amplitude of the surface wave (migrating from the transmitting antenna to the receiving antenna across the surface of the sample, further denoted as DW), the amplitude of the wave reflected from the reinforcement bar RW, and the amplitude of the wave reflected from the metal plate placed under the sample (PW). Additionally, the bidirectional propagation times of each of these waves were analyzed as indicated in Figure 8. It is assumed that the surface wave (DW) parameters depend on the surface properties of the sample (moisture, presence of chlorides). Similarly, the parameters of the wave reflected from the reinforcement (RW) characterize the properties of the concrete throughout the entire thickness of the cover and take into account the influence of corrosion products of the reinforcement bars. The wave reflected from the plate under the sample (PW) characterizes the properties of the concrete throughout the entire cross-section of the sample.

Table 2 summarizes the average values of the aforementioned parameters for non-corroded and corroded samples obtained during scanning with the 2 GHz antenna. As shown, the progress of corrosion had little effect on the surface wave amplitude (Ad+), resulting in only a slight decrease in the Ad− amplitude value (from −3.113 V in the reference samples to −2.988 V in the samples subjected to accelerated corrosion). With the development of corrosion, the standard deviation of the amplitudes Ad+ and Ad− increased slightly, which is associated with a greater spread of the results. A much more significant effect of corrosion processes is observed when analyzing the propagation times of the aforementioned signals (tAd+ and tAd−). With the development of corrosion, the propagation times increased (tAd+ increased from 1.151 to 1.325 ns, and tAd− from 1.418 to 1.585 ns). It should also be noted that the standard deviation of the propagation times increased significantly more than the standard deviation of the amplitudes. However, the value of the standard deviations remains lower for the propagation times. Considering that these values characterize the surface wave, it is presumed that the changes in the wave characteristics are due to the appearance of chlorides in the concrete structure, as well as surface changes resulting from the cyclical freezing and thawing.

From the point of view of corrosion diagnosis, however, a more significant assessment is the evaluation of the parameters of the wave reflected from the reinforcement. As seen in the table below, the samples subjected to accelerated corrosion show a reduced value of Ar+ (3.095 V in the non-corroded samples and 2.859 V in the corroded samples) and Ar− (respectively −2.467 V and −2.319 V). Similarly to before, the samples subjected to accelerated corrosion showed a slightly increased standard deviation of the amplitudes.

In the case of the wave reflected from the reinforcement, a clear extension of the propagation time of the wave, and thus a reduction in its speed, was also recorded. The time tAr+ increased from 1.625 ns to 1.790 ns, and the time tAr− from 1.823 ns to 2.014 ns. Similar to the surface wave, a significant increase in the standard deviation was also observed in this case.

The change in the parameters of the wave reflected from the reinforcement is the result of the simultaneous saturation of the concrete cover with chloride compounds, the presence of corrosion products from the reinforcement migrating to the pores of concrete, as well as structural changes caused by the cyclic freezing and thawing. However, the studies conducted here do not allow for the separation of all these factors and the independent determination of their impact on the wave parameters. This issue will be the subject of separate research.

In order to assess the properties of the wave propagating through the entire cross-section of the sample, the parameters of the wave reflected from the metal plate placed under the sample (amplitude Ap+ and propagation time tAp+) were additionally analyzed. Surprisingly, in the corroded samples, the amplitude value (2.634 V) was slightly higher than in the reference samples (2.609 V). Although this phenomenon can be linked to changes in the concrete structure due to cyclic freezing and thawing, in the authors’ opinion, the increase in the average value of the amplitude is only the effect of the random nature of the local properties of concrete before the development of corrosion. The difference in the values of Ap+ in non-corroded and corroded samples is only 0.025 V and is an order of magnitude smaller than the standard deviation (compare Table 2). It seems, therefore, that the increase in the average value of Ap+ in corroded samples is only a statistical effect.

Additionally, the accelerated corrosion process caused an increase in the standard deviation from 0.304 to 0.370 V. As in the previous cases, in the corroded samples, the wave propagation time increased (from 3.907 to 4.039 ns).

**Figure 9:**
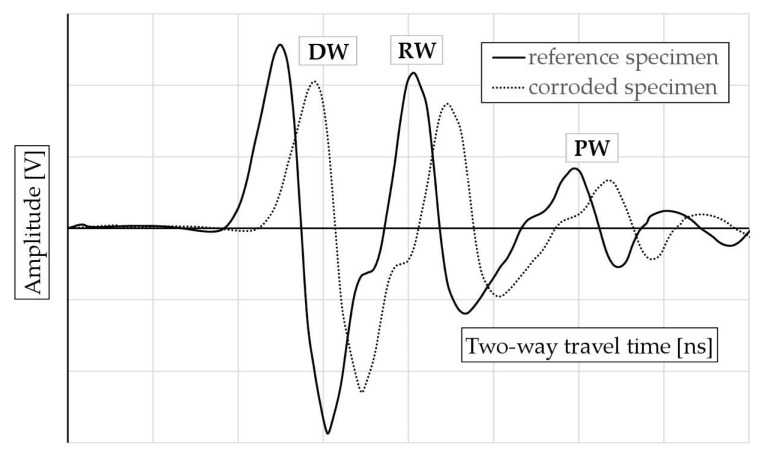
The effect of the corrosive degradation of the tested samples on the reflected wave parameters: DW—surface wave, RW—wave reflected from the reinforcement, PW—wave reflected from the metal plate placed under the sample.

It can be therefore stated that the adopted method for generating accelerated corrosion resulted in changes in the reflected wave parameters, manifested as a decrease in amplitude and an increase in the two-way propagation time. This effect was observed both for the surface wave and for the waves reflected from the reinforcement and the metal plate placed under the sample. The influence of accelerated corrosion on the reflected wave parameters is illustrated in Figure 9.

The changes in wave parameters observed in the tested samples (primarily the decrease in amplitude) seem to be the result of simultaneous changes in the concrete structure due to freezing/thawing, filling of pores and microcracks with chlorides, and the appearance of corrosion products on the surface of the rebar. Although a similar effect may be caused by high humidity of concrete, in the case of the analyzed samples, due to thorough drying and mass control, this is a negligible factor.

In the case of the discussed samples, regardless of the corrosion processes, the presence of chlorides has a significant impact on the decrease in amplitude and the increase in propagation time, which is in accordance with the observations described in [48,49,50,51].

In the scope of corrosion analysis, and especially its early stages, it can be stated that the observed trends are similar to those described in the literature. This concerns primarily the decrease in the amplitude of the wave reflected from the reinforcement. For example, in [52], for concrete samples with a small cover thickness (4 cm), after 1 day of accelerated electrochemical corrosion, a small decrease in the wave amplitude was observed. As indicated by the authors [52], this range corresponds to slight corrosion. However, with more advanced corrosion, an opposite trend was observed, i.e., an increase in the wave amplitude [52].

A decrease in the reflected wave amplitude was also observed in [54]; however, due to the different geometry of the samples, a different method of inducing accelerated corrosion, and different characteristics of the equipment used, it is difficult to directly compare the results. The decrease in amplitude observed in the case discussed here (after 120 freeze–thaw cycles) corresponds to about 3 days of accelerated electrochemical corrosion according to [54]. The ranges of corrosion progress in [54], therefore, seem to be much more advanced than in the described case.

The decrease in wave amplitude cited here as an effect of corrosion was also observed in [56,58].

However, it should be remembered that there are a number of published works in which opposite phenomena were found, i.e., an increase in amplitude and a decrease in wave propagation time with the progress of corrosion, e.g., [57,59]. As mentioned above, these discrepancies are most likely the result of different degrees of corrosion advancement and different phenomena that dominate at different stages of corrosion. Moreover, the authors of published works often do not provide measures of the degree of corrosion advancement that would allow direct comparison of results. The geometry of the samples used and the parameters of the GPR set certainly also have an impact here.

**Figure 10:**
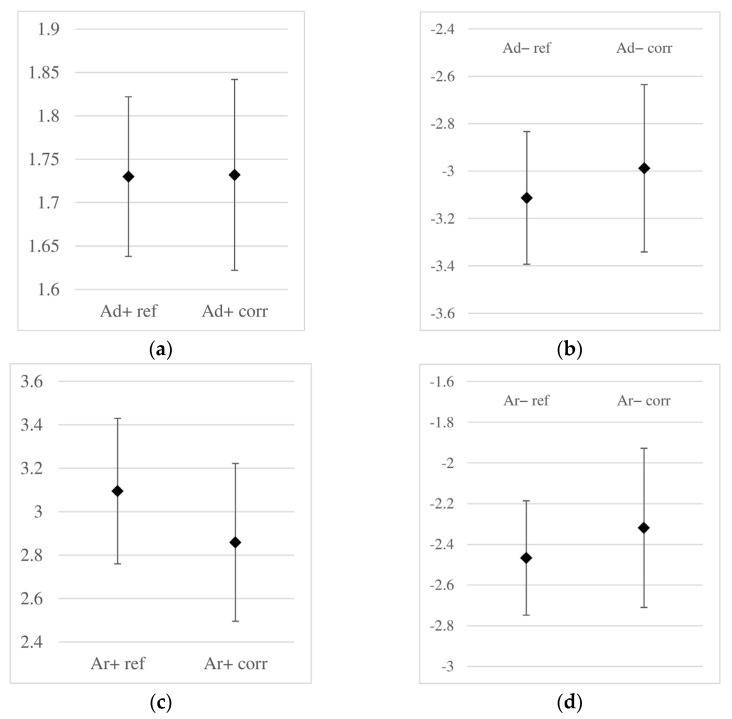
Obtained amplitude ranges [V] of the reflected wave (non-corroded, data with the “ref” index) and corroded (with the “corr” index) samples: (**a**) amplitude Ad+, (**b**) Ad−, (**c**) Ar+, (**d**) Ar− (notations as in Figure 8).

In Figure 10, the averaged maximum amplitudes along with their corresponding standard deviation values are illustrated. On each graph, the amplitude value for the reference samples is plotted on the left side, while on the right side, the amplitude recorded for the sample subjected to accelerated corrosion is shown. As mentioned before, in each case, the accelerated corrosion process caused a slight decrease in amplitude. However, the significant values of the standard deviation resulted in the amplitude ranges for the reference and corroded samples being very similar, making a practical assessment of corrosion occurrence virtually impossible based solely on amplitude analysis.

**Figure 11:**
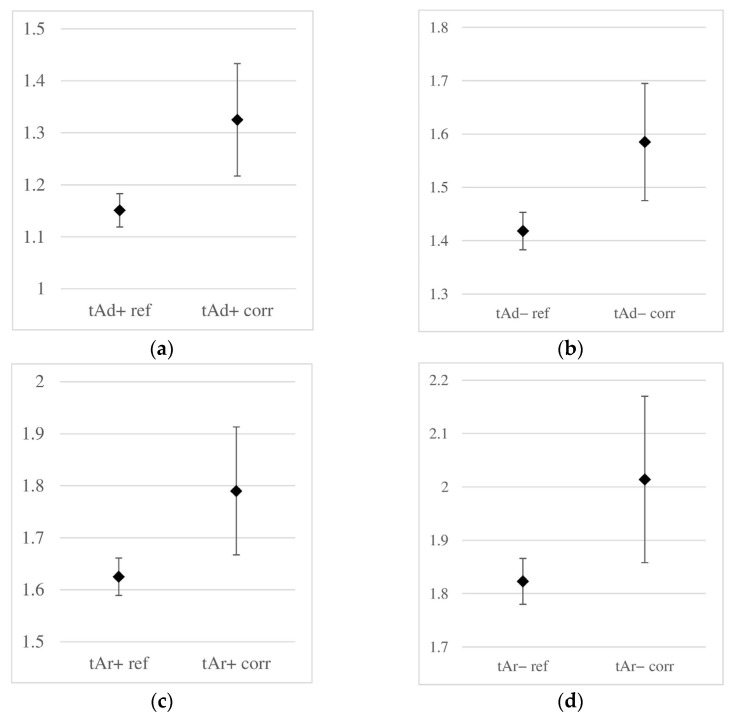
Obtained ranges of two-way propagation times [ns] of the reflected wave in reference (non-corroded, data with the “ref” index) and corroded (with the “corr” index) samples: (**a**) time tAd+, (**b**) tAd−, (**c**) tAr+, (**d**) tAr− (labels according to Figure 8).

A graphical comparison similar to the one above, but for the wave propagation times, is shown in Figure 11. In each case, it was observed that corrosion development was accompanied by an increase in wave propagation time and its standard deviation. However, the standard deviations remain lower than those for the amplitudes, and the ranges of obtained times for corroded and non-corroded samples do not overlap, making it possible to clearly distinguish corroded and non-corroded samples based on the analysis of wave propagation times. Therefore, it can be concluded that in the analysis of corrosion initiation, time is a more reliable parameter than amplitude. However, it should be noted that this remark applies only to the small degree of corrosion advancement analyzed in this study. In the case of further development of corrosion phenomena (such as the formation of cracks in concrete or delamination at the concrete–rebar interface), this relationship may change.

**Figure 12:**
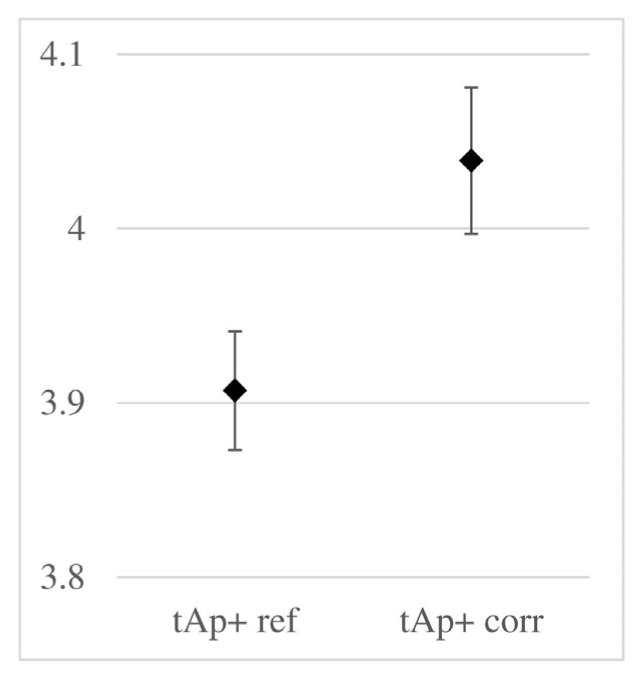
Range of two-way propagation times of the wave reflected from the metal plate beneath the sample (parameter labels according to Figure 8).

Regardless of the above, Figure 12 presents the propagation times of the wave reflected from the metal plate placed beneath the sample.

In the figure above, the difference in average times is relatively small (about 3%), compared to the several percent differences in times observed in the previous figure. Attention is also drawn to the slight increase in the standard deviation in the corroded samples. Both phenomena seem to result from the uneven distribution of chloride content across the cross-section of the sample. In the cover layer, where the chloride concentration is higher, this effect is stronger. On the other hand, Figure 12 illustrates the wave’s migration through the entire cross-section of the sample, including the inner areas, which are less saturated with chlorides.

Table 3 presents the results obtained from measurements with the 900 MHz antenna. A comparative analysis of the amplitudes recorded on reference and corroded samples shows that, in practically no case (surface wave and reflection from reinforcement), accelerated corrosion affected the amplitude value. Similar to the previous case, the standard deviation of the individual amplitudes increased significantly in the corroded samples. The recorded average propagation times also did not change significantly, making the 900 MHz antenna an ineffective tool for corrosion detection. In the case of propagation times, the increase in the standard deviation was relatively small.

## 5. Estimation of Selected Material Properties

Based on the measurement results using the 2 GHz antenna, an attempt was made to determine the derived parameters characterizing the physical properties of concrete in both reference samples and those subjected to accelerated corrosion. The parameters of the wave reflected from the reinforcement were analyzed, so the determined values characterize the cover of the reinforcement bars. It should be noted that the values determined below are averaged over the thickness of the cover. As the depth position changes, the moisture, chloride content in the concrete pores, and the degree of saturation with corrosion products from the reinforcement bars also change.

The wave propagation velocity in the cover was determined using the following formula:

(6) $$v=\frac{2h}{t}$$

where v—mean wave velocity across concrete cover, h—length of the path traveled by the wave in one direction (cover thickness), and t—wave two-way travel time (from transmitter to reinforcement and back to receiver).

In the case of corroded samples, the wave attenuation coefficient was determined relative to the reference (non-corroded) sample:

(7) $$\alpha_{ref}=\left(\frac{20}{h}\right){log}_{10}\left(\frac{A}{A_{ref}}\right)$$

where α_ref_—attenuation coefficient of the wave reflected from the reinforcement relative to the reference (non-corroded) sample, A—amplitude of the wave reflected from the reinforcement of the analyzed sample, and A_ref_—amplitude of the wave reflected from the reinforcement of the reference (non-corroded) sample.

For each of the tested samples, the wave attenuation coefficient relative to the reflection from the metal sheet was determined:

(8) $$\alpha_{plate}=\left(\frac{20}{h}\right){log}_{10}\left(\frac{A}{A_{plate}}\right)$$

where α_plate_—attenuation coefficient of the wave reflected from the reinforcement relative to the metal sheet, A—amplitude of the wave reflected from the reinforcement of the analyzed sample, and A_plate_—amplitude of the wave reflected from the metal sheet.

Additionally, the average dielectric constant of the concrete cover was determined; however, the obtained values should be considered only as approximate. This is due to the complexity of its structure, which consists of both concrete as well as chlorides and steel oxidation products filling its pores. The averaged dielectric constant for all these components was determined based on the three formulas below:

(9) $$\varepsilon_{r}^{1}=\frac{c^{2}}{v^{2}}$$

(10) $$\varepsilon_{r}^{2}={\left(\frac{1+\frac{A_{surface}}{A_{plate}}}{1-\frac{A_{surface}}{A_{plate}}}\right)}^{2}$$

(11) $$\varepsilon_{r}^{3}={\left(\frac{c\;\Delta t\;10^{-9}}{2h}\right)}^{2}$$

In the above formulas, the individual symbols represent ε_r_—dielectric constant of reinforcement cover, c—speed of light in a vacuum (30 cm/ns), A_surface_—amplitude of the wave reflected from the surface of the sample, Δt—two-way propagation time of the wave between the surface of the sample and the reinforcement, and h—thickness of the reinforcement cover (2.5 cm), with the remaining symbols as in the previous formulas.

As follows from the above formulas, $\varepsilon_{r}^{1}$ and $\varepsilon_{r}^{3}$ are averaged values for the entire thickness of the concrete cover, while $\varepsilon_{r}^{2}$ characterizes the surface layer of the concrete.

Table 4 presents the average values of the parameters obtained for the reference and corroded samples.

The average wave velocity in the cover of the reference samples was 10.56 cm/ns. In the samples subjected to accelerated corrosion, this value was 8.61 cm/ns, which represents a decrease of 22.6%. As mentioned before, the decrease in wave velocity (increase in propagation time) is certainly associated with the presence of chlorides in the pores of the cover and the structural changes in the concrete resulting from the cyclic freezing and thawing. However, the conducted research does not provide a definitive answer regarding the influence of the steel corrosion products.

As can be seen from the table, both attenuation coefficients showed a noticeable increase. The value of α_ref_ (attenuation coefficient relative to the reference sample) in the corroded samples was on average −25.5 dB/m. In relation to the parameters of the wave reflected from the metal sheet (100% reflection), the attenuation coefficient α_plate_ in the reference samples was −55.89 dB/m, while in the corroded samples, it was −80.88 dB/m. In the case of both attenuation coefficients, however, very high values of standard deviations are noticeable, as the effect of the significant dispersion of the results. In relation to α_ref_, this effect is additionally intensified by the occurrence of values with different signs (for measurement results in which, contrary to the general trend, the amplitude value in corroded samples was higher than in non-corroded ones, α_ref_ was positive). Since both attenuation coefficients are based on wave amplitude (Equations (7) and (8)), the high values of standard deviations in this case confirm the large variability and unpredictability of the obtained amplitude values, as noted in Section 4.

In the range of dielectric constants, an increase in the values of $\varepsilon_{r}^{1}$ and $\varepsilon_{r}^{3}$ was observed, from 8.14 in the reference samples to 12.33 in the corroded ones. This trend was not observed for $\varepsilon_{r}^{2}$, which, as mentioned, is based on the characteristics of the wave reflected from the surface of the sample and, therefore, does not reflect the phenomena occurring inside the cover (compare the amplitude Ad+ values from Figure 10a). The accelerated corrosion process caused a slight decrease in this value from 7.97 to 7.81 (2%).

## 6. Conclusions

The conducted studies confirmed the applicability of the GPR method for detecting the early stages of reinforcement corrosion development in concrete samples. Unlike other published works, the studies described here were conducted under conditions most similar to natural ones (taking into account the impact of cyclic freezing and thawing on the physical properties of the cover). The obtained results can therefore provide guidance on the use of the GPR method for corrosion detection in real structures. In terms of application conclusions, the following was found:

- In the analyzed range, the study covered two phenomena—the formation of steel corrosion products and the presence of chlorides in the concrete cover. Due to their simultaneous occurrence, it was not possible to separately characterize the impact of these factors on the parameters of the reflected wave;
- The 2 GHz antenna proved to be a useful tool for detecting reinforcement corrosion, while the 900 MHz antenna, due to its low resolution, was unable to capture corrosion phenomena;
- The amplitude of the wave reflected from the reinforcement decreased;
- The propagation time of the wave reflected from the reinforcement increased;
- The propagation time, as a measure of the progress of corrosion processes, is more predictable and can serve as a better basis for drawing conclusions.

Moreover, the research results discussed above allow several conclusions of a cognitive nature to be formulated:

- The development of corrosion did not affect the value of the surface wave amplitude;
- The propagation time of the surface wave increased significantly, likely as a result of the presence of chlorides in the pores of the cover;
- The propagation time of the wave reflected from the metal sheet beneath the sample increased;
- The amplitudes of the reflected wave exhibit significant unpredictability, as evidenced by the high value of the standard deviation.

The obtained results allow for the formulation of the objective and scope of further research, which, in the authors’ opinion, should include the following:

- An evaluation of GPR wave parameters at more advanced stages of corrosion—expansion of corrosion products, delamination at the reinforcement–cover interface, etc.;
- An independent analysis of the impact of the presence of chlorides in the cover and corrosion products on the GPR wave parameters;
- The impact of environmental factors (e.g., concrete moisture) on the measurement results and the ability to assess the intensity of corrosion;
- Correlation of wave parameters with standard measures of corrosion progression;
- The impact of reinforcement diameter and cover thickness on the quality of results and the ability to assess corrosion;
- An analysis of the impact of wave polarization direction.

## Figures and Tables

**Table 1:** The composition of the sample concrete.

Ingredient	Quantity per 1 m^3^
Portland cement CEM I (42.5 N-MSR/NA)	384 kg
Mine sand	680 kg
Basalt aggregate 2–8	600 kg
Basalt aggregate 8–16	650 kg
Tap water	166 L
Plasticizer ADVA Flow 440 (BV/FM)	0.5% (per 1 kg of cement)
Air entrainer Darex AEAW (LP)	0.2% (per 1 kg of cement)

**Table 2:** Comparison of basic characteristics of reflected waves in corroded and non-corroded samples, 2 GHz antenna.

	Non-Corroded Samples		Corroded Samples	
	Value	Std. Deviation	Value	Std. Deviation
Amplitude Ad+ [V]	1.730	0.092	1.732	0.110
Time tAd+ [ns]	1.151	0.032	1.325	0.108
Amplitude Ad− [V]	−3.113	0.280	−2.988	0.353
Time tAd− [ns]	1.418	0.035	1.585	0.110
Amplitude Ar+ [V]	3.095	0.335	2.859	0.363
Time tAr+ [ns]	1.625	0.036	1.790	0.123
Amplitude Ar− [V]	−2.467	0.281	−2.319	0.391
Time tAr− [ns]	1.823	0.043	2.014	0.156
Amplitude Ap+ [V]	2.609	0.304	2.634	0.370
Time tAp+ [ns]	3.907	0.034	4.039	0.042

**Table 3:** Comparison of basic characteristics of the reflected wave in corroded and non-corroded samples, 900 MHz antenna.

	Non-Corroded Samples		Corroded Samples	
	Value	Std. Deviation	Value	Std. Deviation
Amplitude Ad+ [V]	7.695	0.420	7.664	0.586
Time tAd+ [ns]	4.218	0.085	4.216	0.017
Amplitude Ad− [V]	−5.897	0.957	−5.825	1.345
Time tAd− [ns]	4.618	0.055	4.634	0.057
Amplitude Ar+ [V]	7.656	0.608	7.540	0.998
Time tAr+ [ns]	5.014	0.047	5.006	0.050
Amplitude Ar− [V]	−7.384	0.476	−7.558	0.446
Time tAr− [ns]	5.743	0.020	5.752	0.030
Amplitude Ap+ [V]	2.919	0.747	4.182	1.884
Time tAp+ [ns]	6.094	0.021	6.096	0.007

**Table 4:** The values of the material parameters of the samples determined based on the GPR wave analysis.

	Non-Corroded Samples		Corroded Samples	
	Value	Std. Deviation	Value	Std. Deviation
Wave velocity v [cm/ns]	10.56	0.82	8.61	0.78
Wave attenuation relative to the reference sample *α*_ref_ [dB/m]	-	-	−25.5	45.8
Wave attenuation relative to the metal sheet *α*_plate_ [dB/m]	−55.89	38.38	−80.88	45.8
Dielectric constant $\varepsilon_{r}^{1}$	8.14	1.25	12.33	2.25
Dielectric constant $\varepsilon_{r}^{2}$	7.97	1.03	7.81	1.23
Dielectric constant $\varepsilon_{r}^{3}$	8.14	1.25	12.33	2.25

## Data Availability

The original contributions presented in the study are included in the article, further inquiries can be directed to the corresponding author.
